# Meningeomrisiko nach der Behandlung von Krebs im Kindesalter. Eine gepoolte Datenanalyse

**DOI:** 10.1007/s00066-022-02029-7

**Published:** 2022-12-21

**Authors:** Beate Timmermann, Rolf-Dieter Kortmann

**Affiliations:** 1 Klinik für Partikeltherapie, Universitätsklinik Essen, Westdeutsches Protonentherapiezentrum Essen (WPE), Westdeutsches Tumorzentrum (WTZ), Deutsches Konsortium für translationale Krebsforschung (DKTK), Hufelandstr. 55, 45147 Essen, Deutschland; 2 Klinik und Poliklinik für Strahlentherapie, Universitätsklinikum Leipzig, Stephanstrasse 9a, 04103 Leipzig, Deutschland

## Hintergrund

Das Meningeom ist der häufigste Zweittumor nach kranialer Bestrahlung bei Überlebenden von Krebs im Kindesalter, aber es gibt immer noch Unsicherheiten hinsichtlich des Ausmaßes der Dosis-Wirkungs-Beziehung, potenzieller modifizierender Faktoren für das Strahlenrisiko und der Rolle der Chemotherapie.

## Ziel

Bewertung des Meningeomrisikos bei Überlebenden von Krebs im Kindesalter nach Strahlentherapie und Chemotherapie und Identifizierung möglicher modifizierender Faktoren des strahlenassoziierten Risikos.

## Design, Setting und Teilnehmer

Diese internationale Fall-Kontroll-Studie bündelte Daten aus 4 lokalen Fall-Kontroll-Studien von Überlebenden von Krebs im Kindesalter, die zwischen 1942 und 2000 diagnostiziert und bis 2016 nachbeobachtet wurden. Fälle, bei denen ein Meningeom nach Therapie diagnostiziert wurde, wurden als Studienteilnehmer definiert. Eine Matched-pair-Kontrollgruppe wurde nach Geschlecht, Alter bei der ersten Krebsdiagnose und Dauer der Nachsorge gebildet. Die Daten wurden von Juli 2019 bis Juni 2022 analysiert.

## Strahlenexposition

Strahlendosis (Gy) am Ort des Meningeoms und kumulative Chemotherapiedosen, einschließlich intrathekaler und systemischer Methotrexatdosen.

## Methode

Die Hauptauswertungsgröße war die Meningeomentstehung, berechnet aus dem Kreuzproduktverhältnis Odds Ratios (OR) und der entsprechenden Excess Odds Ratios pro Gray (EOR/Gy).

## Ergebnisse

Die Analyse umfasste 273 Überlebende von Krebs im Kindesalter, die ein Meningeom (Fälle) entwickelten, und 738 Überlebende ohne Meningeom (Kontrollen), mit insgesamt 1011 Personen (mittleres [„interquartile range“, IQR] Alter bei der ersten Krebsdiagnose 5,0 [3,0–9,2] Jahre; 599 [59,2 %] weiblich). Die mediane (IQR) Zeit seit dem ersten Tumor betrug 21,5 (15,0–27,0) Jahre. Die Erhöhung der Strahlendosis war mit einem erhöhten Risiko für Meningeome verbunden (EOR/Gy, 1,44; 95 %-CI 0,62–3,61). Es gab keine Hinweise auf eine Abweichung von der Linearität (*P* = 0,90). Im Vergleich zu Überlebenden, die keiner Strahlentherapie ausgesetzt waren, hatten diejenigen, die Dosen von 24 Gy oder mehr erhielten, eine mehr als 30-fach höhere Wahrscheinlichkeit, ein Meningeom zu entwickeln (OR 33,66; 95 %-CI 14,10–80,31). Die Dosis-Wirkungs-Beziehung war bei Patienten, die im Alter von 10 Jahren oder älter behandelt wurden, signifikant niedriger als bei Patienten, die vor dem Alter von 10 Jahren behandelt wurden (EOR/Gy 0,57; 95 %-CI 0,18–1,91 vs. 2,20; 95 %-CI 0,87–6,31; *P* für Heterogenität = 0,03). Das mit Strahlung verbundene Risiko blieb 30 Jahre nach der Exposition signifikant erhöht (EOR/Gy 3,76; 95 %-CI 0,77–29,15). Die Autoren fanden ein erhöhtes Risiko für Meningeome bei Kindern, die Methotrexat erhalten hatten (OR 3,43; 95 %-CI 1,56–7,57), aber keine Hinweise auf eine Dosis-Wirkungs-Beziehung oder Wechselwirkung mit der Strahlendosis.

## Schlussfolgerungen und Relevanz

Diese Ergebnisse deuten darauf hin, dass die Meningen insbesondere bei Kindern, die vor dem 10. Lebensjahr behandelt werden, sehr strahlenempfindlich sind. Die Ergebnisse unterstützen die Reduzierung der Ganzhirnbestrahlung in den letzten Jahrzehnten und die Priorisierung von Ansätzen, die die Strahlenbelastung in gesundem Gewebe für Kinder begrenzen. Die Persistenz eines Risikos für Meningeome auch über 30 Jahre nach kranialer Strahlentherapie könnte dazu beitragen, entsprechende Richtlinien für die Nachsorge zu modifizieren.

## Kommentar

Die Entstehung von Zweittumoren nach Strahlenexposition ist unverändert ein wichtiger Gegenstand der radioonkologischen Forschung, insbesondere in der kurativen Therapie von Tumoren im Kindesalter. Sekundäre ZNS-Tumoren bei Kindern sind dabei besonders relevant, sie stellen den häufigsten Zweittumor nach RT dar [1]. Nach aktuelleren Analysen liegt das Risiko, ein Meningeom zu entwickeln, bei ca. 0,3 % nach 15 Jahren bzw. bei 6 % 40 Jahre nach Primärdiagnose bzw. Therapie, ohne dass ein Plateau zu erkennen ist [1, 2]. Aufgrund des geringen Risikos ist es demzufolge unerlässlich, möglichst umfangreiche Patientenkohorten über einen sehr langen Zeitraum nachzubeobachten. Darüber hinaus sind detaillierte Angaben zur Bestrahlung und den häufig zusätzlichen intensiven systemtherapeutischen Behandlungen notwendig, um zu einer sinnvollen Aussage zu kommen. Diese Anforderungen können die derzeit existierenden nationalen Krebsregister nicht oder nur unzureichend erfüllen. Aus diesem Grunde ist die Initiative, sich mit 4 großen internationalen, renommierten Krebszentren mit umfangreichen Datensätzen zusammenzuschließen, eine notwendige Grundlage, um eine umfassende, detaillierte Datenanalyse durchführen zu können. In der Serie konnte die bereits bekannte Dosis-Wirkungs-Beziehung nach einer kranialen Bestrahlung belegt werden, besonders bei jungen Kindern (unterhalb des 10. Lebensjahrs). Das Risiko für ein Meningeom war, unabhängig von der Bestrahlungsdosis, gegenüber der Kontrollgruppe um den Faktor 11,02 erhöht. Bei Dosierungen über 24 Gy war das Risiko sogar 30-fach erhöht. Die Dosis-Wirkungs-Beziehung war linear. Auch nach Methotrexat war das Risiko für die Entstehung eines Meningeoms erhöht (um den Faktor 3,43). Weitere potenzielle Kofaktoren wie Nachbeobachtungszeitraum und Art anderer zusätzlicher Chemotherapie zeigten in dieser Analyse keinen modifizierenden Einfluss, ohne dass eine Dosis-Wirkungs-Beziehung nachgewiesen werden konnte.

Die Studie hat verschiedene Stärken und Schwächen. Zu den Stärken zählen sicher sowohl die große Untersuchungskohorte, aber auch die sehr gründliche statistische Aufarbeitung der Daten. Als Schwäche ist eine nur abgeschätzte, ortsaufgelöste Dosisrekonstruktion zu nennen, die sicherlich Unsicherheiten birgt. Auch muss postuliert werden, dass bei unterschiedlichen Primärtumoren unterschiedliche Nachsorgekonzepte und Bildgebungen erfolgt sein werden. Unzureichend wurde die Latenzzeit des Auftretens eines Meningeoms in Abhängigkeit von der Strahlendosis untersucht. In der Publikation von Müller et al. (2012) wurden mittlere Latenzzeiten zwischen 10 und 24 Jahren, nach niedrigen Dosierungen zwischen 35 und 48 Jahren zitiert [3]. Diese Latenzzeiten könnten jedoch im Kleinkindesalter auch deutlich unterschritten werden. Im Register für Meningeome im Kindesalter (HIT-ENDO) waren interessanterweise 3 von 5 Meningeomen atypische bzw. anaplastische Subtypen, was das Ereignis eines sekundären Meningeoms auch in Hinblick auf die Prognose noch bedeutsamer macht [3] Bei Müller et al. hatten alle Kinder übrigens in der Primärtherapie zusätzlich eine intensive Chemotherapie erhalten, ähnlich wie die Kinder in der hier vorliegenden Analyse. In Bezug auf den Einfluss der Chemotherapie bleibt es sicher eine wichtige Frage, inwieweit neue Substanzen sich in Bezug auf Sekundärmalignome auswirken werden. In der hier vorliegenden Analyse wurden nur „klassische“ Chemotherapeutika untersucht, von denen nur Methotrexat einen Einfluss auf das Sekundärmalignomrisiko eines Meningeoms gezeigt hat.

Entscheidend ist aber sicher, dass die hier kommentierte Arbeit wichtige Anhalte dafür liefert, dass gerade bei jungen Kindern auch relativ niedrige Strahlendosen Risiken bergen, die wir nach unseren Möglichkeiten minimieren müssen.

Hierzu kann die Protonentherapie hoffentlich einen wichtigen Beitrag leisten, weswegen insbesondere bei Kindern die Protonentherapie zunehmend eingesetzt wird. Skeptiker hinterfragten immer wieder, ob denn die für die Protonentherapie typische Dosiseinsparung im Mittel- und Niedrigdosisbereich überhaupt einen klinischen Nutzen haben würde. Bei einer Reduzierung der Integraldosis um den Faktor 2–3 durch die Verwendung von Protonen ergaben die Risikoabschätzungen eine Senkung des Zweittumorrisikos um den Faktor 2–15 – in Abhängigkeit von verschiedenen Faktoren wie beispielsweise Zielvolumen oder Alter des Patienten [4, 5].

Aber auch erste klinische Daten ergaben im Rahmen der SEER Database Anhalte dafür, dass sich die Dosis-Volumen-Einsparung durch eine Protonentherapie günstig auf eine reduzierte Zweittumorrate auswirken konnte [6]. Sicherlich am interessantesten in Hinblick auf die Senkung des Zweittumorrisikos durch Protonen im Vergleich zu photonenbasierten Radiotherapietechniken ist die 2020 publizierte Arbeit von Xiang et al. [7]. Hierbei wurden mehr als 450.000 Patienten der National US Cancer Database in Hinblick auf ihre Zweittumorinzidenz untersucht, welche XRT, IMRT oder eine PBT erhalten hatten. Die Inzidenz nach PBT war signifikant niedriger als nach einer RT mit XRT oder IMRT, wohingegen sich die Risiken von einer IMRT vs. einer 3‑D-CRT nicht unterschieden. Bei allen bisher gewonnenen klinischen Belegen wurden nicht nur Kinder, sondern auch Erwachsene in die Untersuchungen eingeschlossen. Auch wurden unterschiedliche Zweittumorarten, unabhängig von der Empfindlichkeit der Gewebetypen, bewertet. Man mag annehmen, dass der Unterschied des Zweittumorrisikos noch größer sein müsste, wenn es sich um Kinder und sehr empfindliche Gewebestrukturen wie offenbar die Meningen handelt.

Mit der vorliegenden Arbeit von Withrow et al. verdichten sich also gerade für kraniale Behandlungen die Hinweise darauf, dass die Protonentherapie für Erkrankungen im Kindesalter einen hohen Stellenwert hat. Hierfür ist das Ergebnis der Autoren, dass die Hirnhäute zu den strahlenempfindlichsten Geweben gehören, insbesondere bei Kindern unter 10 Jahren, ein wichtiges Argument. Die Dosierungen bei der (Ganz‑)Hirnbestrahlung sollten so weit wie möglich abgesenkt werden, und es sind Strahlentherapieansätze zu verwenden, die die Exposition von gesundem Gewebe begrenzen. Die Arbeitsgemeinschaft für Pädiatrische Radioonkologie (APRO) und die Deutsche Gesellschaft für Radioonkologie (DEGRO) haben folgerichtig die Rolle der Protonentherapie für kindliche Tumorerkrankungen hervorgehoben und die Kindertumoren im kurativen Therapiekonzept als gesicherte Indikation für die Nutzung der Protonentherapie genannt [8, 9]. Ebenfalls muss aber der Hinweis der Autoren ernst genommen werden, dass aufgrund des Auftretens von Meningeomen als Zweittumoren auch nach 30 Jahren eine entsprechende Berücksichtigung in der Anpassung der Nachsorgeprogramme und der nachhaltigen Patientenaufklärung erforderlich ist, für die wir Strahlentherapeuten eine große Mitverantwortung haben.

## Fazit

*Die bekannte Dosis-Wirkungs-Beziehung bei der Entstehung eines strahleninduzierten Meningeoms konnte innerhalb einer umfangreichen und detaillierten, kooperativen Studie mit 4 großen Zentren nachgewiesen werden. Die bekannten langen Latenzzeiten konnten belegt werden. Das tatsächliche Risiko für ein Meningeom als Zweittumor im Niedrigdosisbereich kann nach dieser Analyse nicht exakt abgeschätzt werden. Bei der hier nachgewiesenen Dosis-Wirkungs-Beziehung vorwiegend bei Kindern unter 10 Jahren empfiehlt es sich, Bestrahlungstechniken einzusetzen, die optimal eine Reduktion der integralen Dosis im Cerebrum erreichen. Hierbei nimmt natürlicherweise die Nutzung der Protonentherapie gerade bei jungen Kindern mit ZNS-Tumor eine zunehmend zentrale Rolle ein, worauf die APRO und DEGRO bereits in ihren Positionspapieren hingewiesen haben. Trotz zahlreicher Schwächen dieser Analyse werden die Daten eine wesentliche Säule bilden, an der sich zukünftige Studien und Nachsorgeprojekte orientieren werden. Die Rolle der Systemtherapien, insbesondere der neuen Substanzen, bleibt noch zu klären.*
